# Accumulation of Deficits as a Proxy Measure of Aging

**DOI:** 10.1100/tsw.2001.58

**Published:** 2001-08-08

**Authors:** Arnold B. Mitnitski, Alexander J. Mogilner, Kenneth Rockwood

**Affiliations:** Department of Mechanical Engineering, Ecole Polytechnique, Montreal, Quebec, Canada H3C 3A7,

**Keywords:** aging, frailty index, health status appraisal, survival, reliability, gamma distribution, biological age, senescence, age-related diseases, relative diversity, macroscopic variable, databases, mathematical modeling

## Abstract

This paper develops a method for appraising health status in elderly people. A frailty index was defined as the proportion of accumulated deficits (symptoms, signs, functional impairments, and laboratory abnormalities). It serves as an individual state variable, reflecting severity of illness and proximity to death. In a representative database of elderly Canadians we found that deficits accumulated at 3% per year, and show a gamma distribution, typical for systems with redundant components that can be used in case of failure of a given subsystem. Of note, the slope of the index is insensitive to the individual nature of the deficits, and serves as an important prognostic factor for life expectancy. The formula for estimating an individual's life span given the frailty index value is presented. For different patterns of cognitive impairments the average within-group index value increases with the severity of the cognitive impairment, and the relative variability of the index is significantly reduced. Finally, the statistical distribution of the frailty index sharply differs between well groups (gamma distribution) and morbid groups (normal distribution). This pattern reflects an increase in uncompensated deficits in impaired organisms, which would lead to illness of various etiologies, and ultimately to increased mortality. The accumulation of deficits is as an example of a macroscopic variable, i.e., one that reflects general properties of aging at the level of the whole organism rather than any given functional deficiency. In consequence, we propose that it may be used as a proxy measure of aging.

### INTRODUCTION

Chronological age is an important determinant of health and survival. Nevertheless, individuals of the same chronological age can differ dramatically from each other in respect to their health status[1]. Indeed, the older the group, the more variance it shows in health. How to characterize individual health status is not clear. Models of genetic heterogeneity can be incorporated in simulations which include demographic data[2,3,4], but precisely how to operationalize such concepts, at the individual level is not known. We have examined the aging process as one of the accumulation of deficits which, while age related, are not usually known as risks for diminished life expectancy, e.g., impaired vision or hearing, or skin problems[5,6]. A quantitative measure, the *frailty index* ($q$), elucidated the accumulation of deficits and is proposed as a means of assessing individual aging. We now consider the following properties of this approach to assessing individual aging, and relative fitness and frailty: the statistical distribution of $q$, progression of frailty with chronological age, and morbidity. In addition, given that in an earlier analysis the rate of decline estimated by a restricted frailty index (one made up of only 20 variables) was indifferent to the variables which made up a given level of impairment, we now investigate the sensitivity of $q$ to the variations in the set of deficits, the value of the frailty index as a predictor of mortality and the type of statistical distributions which represent successful aging and disease states.

### METHODS

The data come from the cross-sectional and longitudinal components of the Canadian Study of Heath and Aging, an investigation of the distribution, risks and burden of dementia in elderly people in Canada[7,8]. The sample was representative of elderly Canadians, and was screened using a standard cognitive test[9]. Those scoring below an impairment cut-point, and a random sample of those scoring above the cut-point were invited to a standard clinical examination[10]. The mean age of this clinical sample was 82.0 years, SD 7.43, range 65–106. 18.7% were in the age group 65–74, 46.3% were aged 75–84 and 34.9% were aged 85+. Date of death was recorded for 1468 participants. The median time to death was 51.9 months for the cohort. In an earlier analysis we selected 20 variables from total 400 variables (for simplicity only binary deficits [Yes/ No] were considered so that each individual was represented by a binary array of 1s and 0s) based on their suspected clinical relevance and statistical properties, e.g., minimum of missing data and maximum of variance across the cases [5,6]. Here we extend our analysis to all available binary variables from the database. This consisted of 92 items, listed in the Appendix. The twenty previously used variables[5] are marked with stars. Items in the frailty index include symptoms (e.g., changes in sleep, memory complaints, low mood), signs (e.g., tremor, decreased peripheral pulses), abnormal laboratory values (e.g., urea, creatinine, calcium), disease classifications (e.g., diabetes mellitus, Parkinson's disease), and disabilities (e.g., dependence in bathing or dressing). Jointly we refer to them as deficits. Note that the deficits cross a range of severity, from items associated with an increased risk of death (e.g., cancer) to those which typically cause more discomfort than disability (e.g., skin problems, constipation). Since some missing data occurred for at least one item and most records, the proportion of deficits incurred by an individual was considered as a measure of health status and is referred to as a frailty index *(q).* Thus, for example, if an individual was given 80 tests from the 92 (12 were not performed and thus considered as missing) and 16 from 80 variables were present, the frailty index is 16/80 = 0.2. Defined in such a way, the frailty index can be easily assessed from any database containing binary deficits by counting the deficits presented in the individual and dividing it by the number of deficits taken into consideration.

### RESULTS AND DISCUSSION

#### Statistical Distributions of Frailty Index

Let us first consider the distribution of frailty across all individuals represented in the CSHA database. In Fig. 1, the distribution of $q$ is shown for 2913 individuals This distribution is well represented by the gamma density function (solid line) according to the formula:

$$f(q) = \lambda^k q^{k-1} e^{-\lambda q} / \Gamma(k) \qquad (1)$$

where $\lambda$ and $k$ are the scale and shape parameters and $\Gamma(k)$ is the gamma function. The mean $\mu$ and variance $\sigma^2$ of the distribution are related to the shape and scale parameters, $\lambda = \mu/\sigma^2$ and $k = (\mu/\sigma)^2$, respectively. The parameters of gamma distribution were estimated: $k$ = 2.2 and $\lambda$ = 10.638 and goodness of fit was evaluated in two ways, by using the multiple correlation coefficient between the experimental and theoretical distributions ($r$ = 0.985, corresponding to the 97% explained variance) and by applying the chi-squared test : $\chi^2$ (35) = 21.5 which is much less than the critical value 53.5, $p < 0.05$. The hypothesis of a gamma distribution of $q$ may be thus accepted.

The gamma distribution belongs to the family of distributions (such as log-normal, Weibull, Gompertz, etc.) often used for the modeling of the system's failure in the reliability theory applications and survival analysis[11-13]. Specifically, the gamma distribution occurs for systems with spare, redundant components which can be used in case of failure of a given subsystem[14].

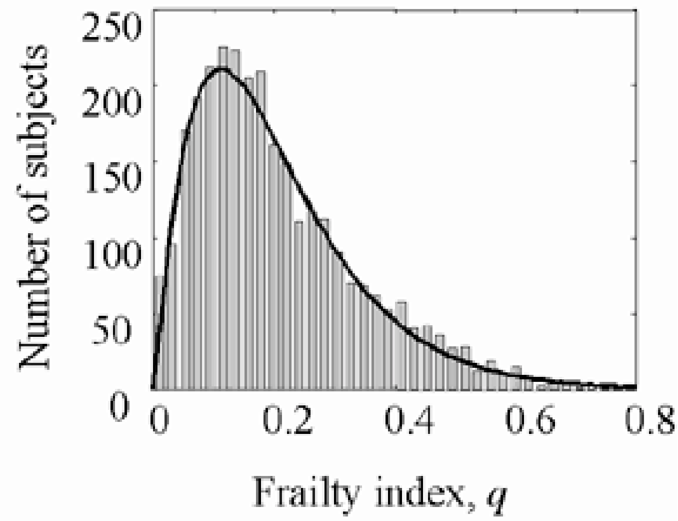

**FIGURE 1.** Distribution of frailty index among 2913 elderly Canadians. The histograms represents the experimental data and the solid curve is a gamma distribution with the parameters of scale (10.638) and shape (2.2).

The shape parameter of the gamma distribution is close to 2, corresponding to a system with double redundancies. The process of accumulation of deficits in individuals therefore may be approximated as a two-stage process, where the first stage corresponds to the organism's compensatory abilities being sufficient to resist the pressure of the environment. Later, destructive processes are predominant corresponding to the decline of the function with age. The gamma distribution found from our analysis distinctively coincides with that suggested from theoretical considerations[2] of addressing the heterogeneity of frailty in order to explain important peculiarities of demographic data. The substantial difference is, however, that our definition of the *frailty index* is based exclusively on the experimental data, and assessable individual characteristics, and not on a 'post hoc' mortality analysis. Since relative frailty can be assessed, in principle, for any individual, this may have potential in other clinical applications.

At this stage we analysed the distribution of frailty without distinguishing between different ages, in part because relatively poor statistics (100 cases in average for each age) would compromise the precision of fit. On the other hand, most (80%) of the CSHA clinical sample is between 70 and 90 years old, and thus may be considered as relatively homogeneous. Nevertheless, the evolution of the statistical distribution with age could be of significant interest, especially if wider ranges of ages with more cases for each age group become available.

### Accumulation of Deficits with Age

Let us consider now the progression of frailty averaged across individuals at each age, $t$, $q(t)$ ($0 < q < 1$). The correlation coefficient between age and $q$ across all (2913) individuals, $r(t,q(t)) = 0.241$. In consequence, as a first approximation, we can accept the hypothesis of a linear accumulation of deficits with age ($p < 0.01$). However, the logarithm of frailty index gives a significantly ($p < 0.05$) greater value of the correlation coefficient, $r(t,\ln(q)) = 0.290$. By averaging the logarithms of $q$ across the individuals with the same age, $t$, we arrived at the equation:

$$\ln(q) = -4.62 + 0.033 \cdot t \qquad (2)$$

where $66 \le t \le 102$ and the correlation coefficient $r = 0.964$. According to Equation (2) the average level of accumulation of deficits increases exponentially with age $t$. In Fig. 2, the points correspond to the value of $q$ averaged across individuals at the same age, $t$, and the solid line corresponds to the exponential function produced from Equation (2), $q(t) = 0.01 \cdot \exp(0.033 \cdot t)$.

Therefore the average level of frailty significantly increases with age, at a rate of 3% per year, as we have reported earlier, using a frailty index of 20 items[5]. We were interested to know whether this relationship was particular to the values that make up the frailty index, i.e., to know whether this rate depends on how many and which deficits are taken into account. Therefore we generated the random samples of items with different power. To do so we checked samples of each of 60, 40, 30, and 20 items, randomly selected at each age, and repeated the calculation several times. The number of re-samplings varied from 10 to 40 depending on the number of items; the lower the number of items, the greater the number of repetitions applied. The parameters of regressions produced in the random simulations (after averaging the parameters across random trials) were close to the values presented in Equation (2). For example, when the index was defined from 60 randomly deficits, we obtained the regression equation, $\ln(q) = -4.41 + 0.032 \cdot t$ ($r = 0.940$, with standard errors for the intercept and slope of 0.15 and 0.002, respectively), and for 40 items used for the frailty index the parameters were found close to the previous values, $\ln(q) = -4.59 + 0.033 \cdot t$ ($r = 0.935$). Frailty, defined as a proportion of deficits appears to be a robust characteristic, and not sensitive to the choice of particular items.

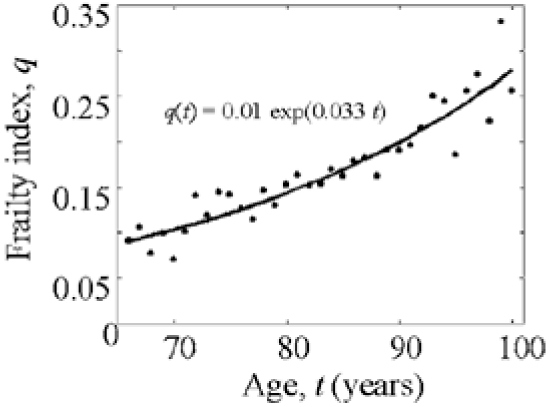

**FIGURE 2.** Accumulation of the frailty index with chronological age. Points represent the proportion of deficits averaged across the individuals with the same age. Solid lines represents exponential function obtained from to the least square regression, according to Equation (2).

That the index is robust likely reflects redundancy in the variables[15]. This redundancy is a consequence of strong interrelationships between variables, and likely reflects the numerous connections between different elements and sub-systems of the human organism[5,15]. Roughly speaking, each variable is related, at least in part, to many others. Redundancy in relation to reliability and aging has been thoroughly discussed[12] and likely also reflects the earlier concept of the reliable system arising from nonreliable elements[16,17].

The redundancy of deficits is a statistical phenomenon[5] and not a functional one (in a mathematical sense). Each variable is linked to many others, but still has its own unique dimension. For example, consider plasma glucose concentration and diabetes mellitus. We expect them to be redundant, which they are, but only to a certain degree. In the CSHA database, 57 individuals were diagnosed with diabetes mellitus and with a high glucose level, but 67 individuals with diabetes had a normal plasma glucose. Thus the two variables are not absolutely redundant: some people with diabetes experience better control than others. Similarly, 71 individuals with significantly high levels of plasma glucose were not diagnosed with diabetes. Thus, overall, the frailty index appears to be robust because it is based on a count of deficits, each of which, in turn, is related to others. The rate of increase in the accumulation of deficits may be, therefore, regarded as an estimate of the rate of aging, at least as a first approximation. (Of interest, it has been noted that the rate of aging is the rate of irreversible elementary failures[12].) Since the average level of accumulation of deficits increases monotonically with age, it can be considered as a proxy measure of aging. At the individual level, the individual rate of accumulation of deficits (which may be easily assessed from the list of available deficits) may be regarded as an individual rate of aging. Since deficits accumulate faster for individuals from unwell groups (in this case using cognitive syndromes as examples of wellness and disease) than for average individuals with no cognitive impairment, one can say that the individuals from unwell groups are aging faster compared to average. In the other words, their biological age exceeds their chronological age. We have proposed that the difference between the two is a precise estimate of relative fitness (where an individual's personal biological age (PBA) is less than his/her chronological age) or frailty (where an individual's PBA is greater than his/her chronological age)[5].

### Frailty Index and Survival Probability

Chronological age is commonly used as an essential predictor of mortality. Calculating the correlation coefficient between time to death, $T$ (available for 1468 individuals of the cohort over five years period from the longitudinal component of CSHA-2 database) and age, $t$, we found $r(T, t)$ = -0.088 which is a small but statistically significant value, $p < 0.05$. However, time to death was found more closely correlated with frailty index, $r(T, q(t))$ = –0.234 ($p < 0.0001$). This indicates that the value of the frailty index offers a significant advantage in predicting mortality compared to age. Earlier we demonstrated that the frailty index reflected an individual's health status and better predicts mortality than chronological age[5]. We have established that people at the same age are more likely to die if their frailty index is greater[5].Taking from CSHA database the information about survival time in the cohort over the five year period, we analyzed how the accumulation of deficits may be used as a predictor of survival. Let $T_i$ be time to death for the $i$-th individual and $P(q_i,T_i)$ be the probability that $i$-th individual will survive during the period $T_i$, given, $q_i$. The probability ($P$) that an individual with a given $q$ will survive $T$ months or more is a function of two variables $q$ and $T$. Since $P$ evidently diminishes when both $q$ and $T$ increases, one can expect that $P$ may depend more closely on the product $q{\cdot}T$ than from each multiplier separately. Let us introduce $V$ as product $V = q{\cdot}T$ (mortality product, since mortality increases with $V$) and consider survival probability as a function of single variable $V$. In Fig.3, the density function of the survival probability is shown. Histogram represents the experimental data (1468 cases). The solid line corresponds to the gamma density function, $f(V)$ :

$$f(V) = \lambda^k V^{k-1} e^{-\lambda V} / \Gamma(k) \qquad (3)$$

with the parameters $k$, $\lambda$ related to the mean $\mu$ and variance $\sigma^2$ : $\lambda = \mu/\sigma^2$, and $k = (\mu/\sigma)^2$. We obtained the estimates of $\lambda$ and $k$ ($k$ = 1.456 and $\lambda$ = 0.209). Goodness of fit was assessed by the multiple correlation coefficient between the experimental data and estimated values of 0.998 corresponding to 99% of the explained variance, and ($\chi^2$ (29) = 28.4 $p < 0.05$). Consequently, the hypothesis that the mortality product has a gamma distribution can be accepted. The survival probability $P(V) = \Pr\{V \geq q{\cdot}T\}$ may be estimated using the tail of the incomplete gamma function

$$\Pr\{V \geq q \cdot T\} = 1 - I_k(q{\cdot}T) = \int_{qT}^{\infty} \lambda^k \cdot V^{k-1} \cdot e^{-\lambda V} \cdot dV/\Gamma(k) \qquad (4)$$

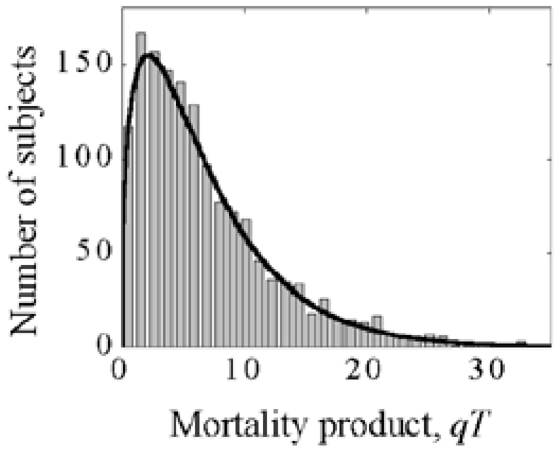

**FIGURE 3.** Statistical distribution of the mortality product among 1468 seniors. The histograms represents the experimental data and the curve is a gamma distribution with the parameters of scale (0.209) and shape (1.456).

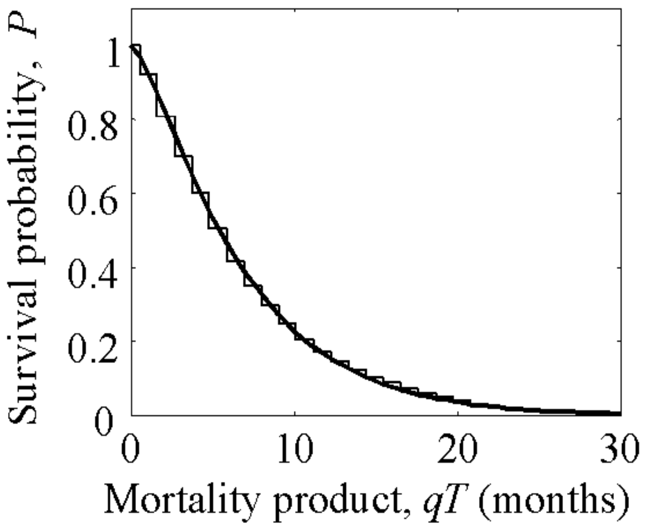

**FIGURE 4**. Survival probability as a function of the mortality product *V*. Stair graph represents the proportion of survived individuals given the product of the frailty index and time to death calculated from the database and the curve corresponds to Equation (4).

In Fig. 4, the graph corresponds to the empirical value of the survival probability and the solid line corresponds to the cumulative survival function (4) with the parameters of $k = 1.456$ and $\lambda = 0.209$), providing an excellent fit ($r = 0.999$).

In contrast with the conventional approach, where survival probability is estimated as a function of chronological age, we regarded the survival probability as a function of the current status defined by the accumulation of deficits at the moment of appraisal. Formula (4) gives the relationships between 3 variables, the survival probability *P*, the frailty index, *q* and life expectancy *T* at the time of appraisal. Each of these values may be calculated given two others. For example, it is possible to determine the probability of surviving for *T* months given the accumulation of deficits, *q*. For example, for an individual with $q = 0.20$, the chance of surviving at least 2 years (24 months, thus $V = 0.2 \cdot 24 = 4.8$) equals 0.55 (55%), and for 5 years ($V = 0.2 \cdot 60 = 12$) is 0.16 (16%).

As noted, the deficits include many which customarily are considered non-vital, such as hearing impairment or loss of vision. Our finding that the proportion of deficits influences life expectancy is consistent with vulnerability in any complex system arising due to loss of interconnectedness of the part[15-18]. We suggest that this correspond to the clinical syndrome of frailty, in which there is considerable homogeneity in clinical patterns of multi-system failure, in contrast to the usual clinical conceptualization that relies on system-specific illness presentation[1,19].

### Frailty Index in Successful Aging and Senescence

As described, we found a gamma fit for the statistical distribution of the accumulation of deficits for the whole data. Of further interest is the distribution of the frailty index for different diagnostic groups. Table 1 shows the parameters of the distributions (mean and standard deviation) for 15 diagnostic groups, together with the type of the statistical distribution best fitted by the experimental histogram.

**TABLE 1**
**Mean Frailty Index (μ) Standard Deviation (σ) and Coefficient of Variation (σ/μ) for Different Diagnostic Groups**

| Diagnosis | N | Mean (μ) | STD (σ) | σ/μ | Distribution |
|---|---|---|---|---|---|
| No cognitive impairment (NCI) | 921 | 0.106 | 0.065 | 0.613 | Gamma |
| Cognitive impairment, no dementia (CIND) | 861 | 0.167 | 0.090 | 0.539 | Gamma |
| Mild probable Alzheimer's Disease | 86 | 0.176 | 0.085 | 0.483 | Normal |
| Moderate probable Alzheimer's Disease | 163 | 0.218 | 0.095 | 0.436 | Normal |
| Severe probable Alzheimer's Disease | 199 | 0.394 | 0.123 | 0.312 | Normal |
| Possible Alzheimer's dementia Atypical | 35 | 0.199 | 0.100 | 0.502 | Normal |
| Possible AD vascular | 137 | 0.321 | 0.131 | 0.408 | Normal |
| Possible AD and Parkinson's Dementia | 31 | 0.387 | 0.138 | 0.356 | Normal |
| Possible AD coexisting Dementias | 98 | 0.271 | 0.135 | 0.499 | Normal |
| Mild vascular dementia | 51 | 0.256 | 0.110 | 0.429 | Normal |
| Moderate vascular dementia | 85 | 0.331 | 0.106 | 0.320 | Normal |
| Severe vascular dementia | 72 | 0.447 | 0.125 | 0.279 | Normal |
| Parkinson's dementia | 27 | 0.370 | 0.112 | 0.302 | Normal |
| Other dementias | 52 | 0.288 | 0.125 | 0.434 | Normal |
| Unclassified dementias | 96 | 0.279 | 0.118 | 0.423 | Normal |

Table 1 also demonstrates that the means and standard deviations are not independent. The relationship between those two estimated parameters is revealed in Fig. 5, with coefficients of variation, σ/μ, as a function of μ such that,

$$\frac{\sigma}{\mu} = 0.689 - 0.944 \cdot \mu \qquad (5)$$

the correlation coefficient r = –0.928, corresponding to about 86% of the explained variance. The variation coefficient may be regarded as the relative within group variability (characterized by the standard deviation). As such, the relative variability (or in other words, *diversity*) σ/μ monotonically decreases as deficits accumulate.

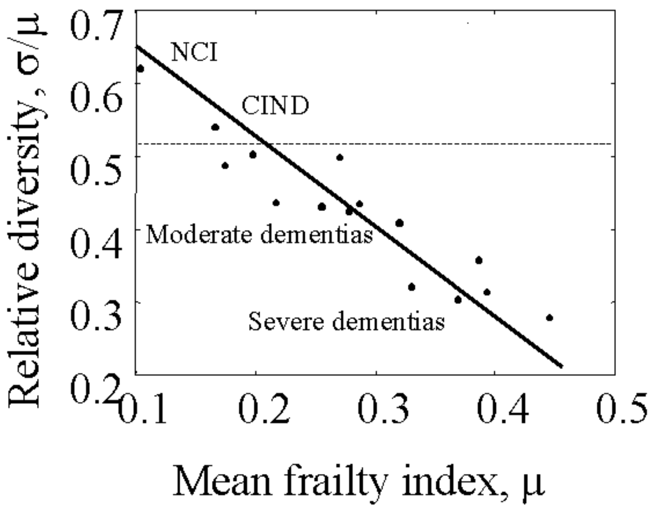

**FIGURE 5.** Reduction of the variation coefficient (relative variability) for different diagnostic groups with severity of impairment. Points represent estimated values of the parameters and solid line represents the regression according to Equation (5). Dashed line separates the successful nonpathological groups (gamma distributed) from morbidities (normally distributed).

As in Fig. 5, relative diversity is diminished by as much as two times across diagnostic groups. In other words, diversity reduction is seen with increasing severity of dementia. Based on an earlier analysis, using a more narrowly defined frailty index, we have proposed that this can be interpreted as indicating elimination from the population (due to death) of those individuals with the greatest number of deficits[19]. In Fig. 5, the dashed line corresponding to the value of the relative diversity slightly above 0.5 separates morbidities from normal aging groups. A reduction of diversity is equivalent to an increase of *uniformity*. Note that more uniform or regular behavior of complex dynamical systems corresponds to a loss of integration of a system approaching its failure[18,20] Interestingly, the other indicator of disintegration and loss of complexity was described earlier in terms of deterioration of normally synergetic relationships between different deficits for different forms of dementia[15].

The most important difference between well and unwell groups is reflected in the change of statistical distribution of the accumulation of deficits (Table 1, right column). Fig. 6 provides illustrative examples for the successful aging and for severe Alzheimer's disease groups. One can see that for the successful aging groups, the statistical distribution of frailty index is the gamma distribution ($\chi^2(10) = 3.89$; $p < 0.05$). Such distribution indicates the damage control or/and prevention mechanisms. In contrast, for the unwell groups, a normal distribution was found ($\chi^2(12) = 6.44$; $p < 0.05$). This corresponds to systems with a large number of independent and uncompensated failures of subsystems and elements. In other words, the coordination of the different systems is defected, as manifested in the independent accumulation of different deficits. Interestingly, there is no significant difference in the distributions of age; each of the 15 age groups has a normal distribution. We have observed a loss of statistical relationships between different deficits for the unwell groups in an earlier analysis[15] as well as the change in the type of the distributions between well and unwell groups[21]. This reflects a failure in mechanisms of damage control normally present in successfully aging groups. The difference between those two distributions is of some interest, and may point to an important difference between aging and senescence. The latter is said to be "the prelude to death from 'old age' "[18].

The fact that a gamma distribution was found only for well groups is consistent with the view that, during aging, processes of damage control or damage prevention are essential characteristics of the organisms, whatever the specific mechanisms of such control[22-30].

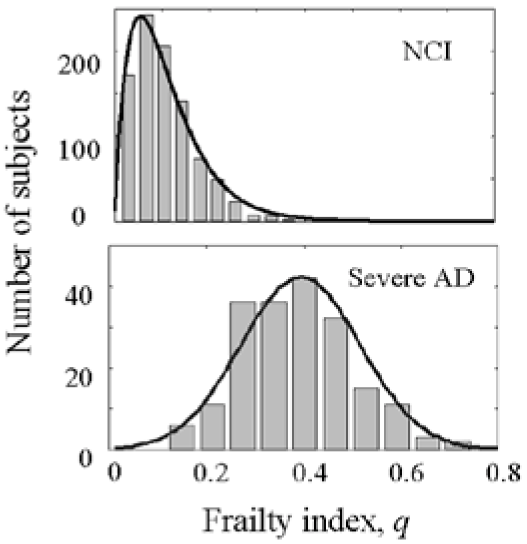

**FIGURE 6.** Distribution of frailty index for normal (NCI) group (A) and Alzheimer's severe dementia (B). The histograms represent the experimental data and the curves are a gamma distribution (A) with mean 0.106 and standard deviation 0.065, and normal distribution (B) with mean 0.394 and standard deviation 0.123.

### CONCLUSIONS

We introduced a frailty index as the proportion of deficits present in the individuals at the time of their health appraisal. It was hypothesized that the index value may reflect health status of the individual and groups of individuals, so as to be highest for individuals from the unwell groups. It was first demonstrated that the gamma distribution (known in the reliability theory and its applications) significantly represents the experimental statistical distribution of the accumulation of deficits across all individuals from a representative database of elderly Canadians. Secondly, the accumulation of deficits was shown to increase monotonically with chronological age. The re-sampling procedure with different sets of deficits demonstrate that the accumulation of deficits is a robust characteristic with the slope that is not sensitive to the choice of particular items. Third, the survival analysis based on the cohort over a 5-year period supported the hypothesis that survival probability can be estimated from the value of the frailty index without reference to chronological age. Fourth, the analysis of the frailty index for well and unwell groups revealed that the relative diversity of the accumulation of deficits decreases with the severity of the impairment. Finally, two different types of statistical distributions were found. The gamma distribution was found for the successful aging group, indicating that during aging the processes of damage control/prevention are essential characteristics of the organisms. In contrast, for the unwell groups, the normal distribution was found corresponding to systems with a large number of independent and uncompensated failures of subsystems and elements. The difference in those distributions reflects the fundamental difference between successful aging and senescence.

On these grounds we propose that the frailty index can be used as a proxy measure of aging and mortality. The index is an example of a *macroscopic variable* which represents general properties of aging for the whole organism (system damage) rather than any particular functional deficiency or decline. Our definition of the frailty index implies equality of the deficits. While this is a useful first approximation, and while it may hold for groups (as a reflection of frailty) at the individual level its applicability is less certain. Weighing schema may be of more use in that regard. Finally, we had at our disposal data only for elderly people. How this may work with a more broadly representative sample is not clear, but the approach may hold merit in being able to estimate preclinical disease status. We look forward to being able to address these points in subsequent investigations.

### ACKNOWLEDGMENT

The data reported in this article were collected as part of the Canadian Study of Health and Aging. The core study was funded by the Seniors' Independence Research Program, through the National Health Research and Development Program (NHRDP) of Health Canada (Project No. 6606-3954-MC(S)). Authors also thank the anonymous referees for their comments.

## APPENDIX

### List of Variables*

| | | |
|---|---|---|
| 1 | CLOUDING | clouding/delirium |
| 2* | SLEEPCH | sleep changes |
| 3* | MOBILITY | mobility impairment |
| 4* | MEMORY | difficulty with memory |
| 5 | MOOD | difficulty with mood |
| 6* | GOUOUT | difficulty with going out |
| 7* | COOKING | difficulty with cooking |
| 8* | GETDRES | difficulty with getting dressed |
| 9* | GROOM | difficulty with grooming |
| 10* | BATH | difficulty with bath |
| 11* | TOILET | difficulty with toileting |
| 12 | URINE | incontinence of urine |
| 13 | STOOL | incontinence of stool |
| 14 | ONSET | onset of symptoms (gradual or abrupt) |
| 15 | SAD | feel sad, blue or depressed |
| 16 | REST | resting tremor |
| 17 | ACTION | action tremor |
| 18 | CHOREA | dyskinesias/chorea |
| 19 | AKINESIA | akinesia |
| 20 | HXSTROKE | history of stroke |
| 21 | HEADACHE | headaches of recent onset |
| 22* | LOSSVISI | chronic visual loss |
| 23* | LOSSHEAR | difficulties with hearing |
| 24* | ARTERIAL | arterial hypertension |
| 25 | CARDIAC | cardiac symptoms |
| 26 | RESPIRAT | respiratory complaints |
| 27 | MALIGNAN | history of malignancy |
| 28* | GASTRO | gastro-intestinal complaints |
| 29* | URINARY | urinary complaints |
| 30 | HISTHYRO | history of thyroid disease |
| 31* | HXDM | history of diabetes mellitus |
| 32 | NECH | physical exam: head and neck (normal, abnormal) |
| 33 | THYROID | physical exam: thyroid |
| 34 | BREAST | physical exam: breast |
| 35 | LUNG | physical exam: lungs |
| 36* | VASCULAR | physical exam: cardiovascular |
| 37 | CAROTIDS | physical exam: peripheral pulses |
| 38 | ABDOMEN | physical exam: abdomen |
| 39 | RECTUM | physical exam: rectum |
| 40* | SKINCLIN | physical exam: skin |
| 41 | SUCKING | neur exam: sucking, release sign |
| 42 | SNOUT | neur exam: snout, release sign |
| 43 | PALMOMR | neur exam: palmomentals R, release sign |
| 44 | BULK | neur exam: bulk |
| 45 | TONENECK | neur exam: tone/neck |
| 46 | TONELIMB | neur exam: rone limb |
| 47 | TREMORRE | neur exam: tremor/rest |

* Twenty variables which previously were used for the derivation of frailty index[5] are marked with asterisks.

| | | | |
|---|---|---|---|
| 48* | TREMORAC | neur exam: | tremor/action |
| 49 | MYOCLONM | neur exam: | diskinesia |
| 50 | BRAADFACE | bradykinesia/face | |
| 51 | BRADLIMB | bradykinesia/limb | |
| 52 | COLIMB | coordination/limb | |
| 53 | COTRUNK | coordination/trunk | |
| 54 | POSTURE | posture/standing | |
| 55* | GAIT | gait, motor system | |
| 56* | VIBRAT | vibration, sensory system | |
| 57 | ONSETAGE | onset between ages 40 and 90 | |
| 58 | GLUCOSE | lab: glucose | |
| 59 | SODIUM lab: | sodium | |
| 60 | POTASSIU | lab: potassium | |
| 61 | BUN | lab: BUN | |
| 62 | CREATINI | lab: creatinine | |
| 63 | CALCIUM | lab: calcium | |
| 64 | ALKPHOSP | lab: phosp. | |
| 65 | TSH | lab: TSH | |
| 66 | B12 | lab: B12 | |
| 67 | FOLATE | lab: serum folate | |
| 68 | VDRL | lab: VDRL | |
| 69 | PROTEINlab: | total protein | |
| 70 | ALBUMIN | lab: albumin | |
| 71 | PHOSPHOR | lab: inorganic phosphate | |
| 72 | RBC | lab: RBC folate | |
| 73 | FABSTRUC | impaired abstract thinking | |
| 74 | FJUDGEME | impaired judgement | |
| 75 | FAPHASIA | aphasia | |
| 76 | FAPRAXIA | apraxia | |
| 77 | FAGNOSIA | agnosia | |
| 78 | ADL | | |
| 79 | IADL | | |
| 80 | HBP | high blood pressure | |
| 81 | HEART | heart and circulation problems | |
| 82 | STROKE | stroke or effect of stroke | |
| 83 | EYETROUB | eye trouble | |
| 84 | EARTROUB | ear trouble | |
| 85 | CHEST | chest problems | |
| 86 | BLADDER | lose control of bladder | |
| 87 | BOWELS | lose control of bowels | |
| 88 | DIABETES | diabetes | |
| 89 | KIDNEY | kidney trouble | |
| 90 | PARKINSO | Parkinson’s disease | |
| 91 | RELEASE | release signs | |
| 92 | SINCE | years since onset | |
